# Perinatal depression in Nigeria: perspectives of women, family caregivers and health care providers

**DOI:** 10.1186/s13033-017-0134-6

**Published:** 2017-04-17

**Authors:** Ademola Adeponle, Danielle Groleau, Lola Kola, Laurence J. Kirmayer, Oye Gureje

**Affiliations:** 1 Department of Psychiatry, McGill University, 1033 Pine Avenue West, Montreal, QC H3A 1A1 Canada; 2 Culture and Mental Health Research Unit, Institute of Community and Family Psychiatry, Jewish General Hospital, 4333 Côte-Ste-Catherine Road, Montreal, QC H3T 1E4 Canada; 3 Department of Psychiatry, College of Medicine, University of Ibadan, Ibadan, Nigeria

**Keywords:** Nigeria, Perinatal depression, Qualitative interviews, Illness explanatory models

## Abstract

**Background:**

Perinatal maternal depression is common and undertreated in many sub-Saharan African countries, including Nigeria. While culture shapes the social determinants and expression of depressive symptoms, there is a dearth of research investigating these processes in African contexts.

**Methods:**

To address this gap, we conducted in-depth interviews with 14 women with perinatal depression, 14 of their family caregivers and 11 health providers, using the *McGill Illness Narrative Interview* as part of a larger trial of a stepped-care intervention. Interpretation of themes was guided by cultural constructivist and critical anthropological perspectives that situate perinatal depression in its complexity as a disorder that is embedded in webs of social relations and embodied practices.

**Results:**

Study respondents used idioms of distress that identified perinatal conditions that consist of somatic, affective, cognitive and behavior symptoms found in depressive disorders. Respondents viewed mental health problems in the perinatal period as tied to sociomoral concerns over gender roles and women’s position within the household. Conflict between women’s effort to be assertive to address interpersonal problems, while needing to be seen as non-aggressive contributed to their distress. Causal explanations for depression included husband’s lack of care, family problems, “spiritual attack”, having a female child when a male child was desired, and not resting sufficiently after childbirth. Guilt about breaching social norms for women’s conduct contributed to self blame, and feelings of shame.

**Conclusions:**

Clinical assessment and interventions as well as public health prevention strategies for perinatal depression in global mental health need to consider local social contexts and meanings of depression, which can be explored with narrative-based methods.

**Electronic supplementary material:**

The online version of this article (doi:10.1186/s13033-017-0134-6) contains supplementary material, which is available to authorized users.

## Background

Depression is common among women of child bearing age, with women living in low- and middle-income countries being particularly at high risk [1]. In Nigeria, 10–20% of consecutive attendees in primary care have depression [2], with reported rates of perinatal depression from 10 to 30% [3]. Perinatal maternal depression is associated with adverse fetal and infant outcomes [4]. Despite its high disease burden, perinatal depression remains undertreated in many African countries, including Nigeria [5, 6]. The experience of depression is shaped by local contexts, idioms of distress, and explanatory models [7]. Information about these local meanings and modes of interpretation can help guide effective prevention and treatment interventions [8]. However, there is a paucity of studies that use qualitative methodologies to investigate local cultural understandings of perinatal depression, the lived experience of illness, and the social context of pregnancy and childbirth [9, 10]. To date there are no such studies published from Nigeria.

Previous studies of women’s experience of perinatal depression have largely focused on identifying culture-specific “risk” factors, psychosocial and health factors, contributing to the onset of perinatal depression in Africa. For example, a recent systematic review involving both quantitative and qualitative studies of perinatal depression in sub-Saharan Africa [9] sought to identify cultural “risk” factors for perinatal depression in Africa. The review found unique cultural risk factors to include negative cultural perceptions, including how baby and pregnancy are viewed by others, for example, stigma associated with being a single mother and having a child out of wedlock [11], and having a female child where preference is for a male [11, 12]. Other identified cultural risk factors include difficulty adhering to perinatal traditions, and fears of repercussions if prescribed rites are violated [12]. Another identified cultural risk factor is the view of pregnancy as a period of vulnerability for mother and child, especially to supernatural affliction [10, 12]. The studies reviewed come from such countries as Ethiopia, Gambia, Zambia, Malawi and Tanzania. What has however been missing in the research is work focused on elaborating the processes that may be mediating the identified cultural risk factors.

To address this knowledge gap, we conducted an in-depth qualitative study of the cultural context of perinatal depression in Nigeria. Our approach was informed by cultural constructivist and critical anthropological perspectives to situate perinatal depression in ecosocial context, which assume that: (1) the ways that perinatal depression is understood and responded to by individuals are influenced by local cultural models and interpretive systems; and (2) the illness has complex social ramifications that are not limited to the patient, but include their spouse, newborn baby, other children, and the extended family.

## Methods

### Study design

The current report is based on qualitative interview data from the formative study of the Expanding Care of Perinatal Women with Depression (EXPONATE) research program, a mixed-methods study to assess the effectiveness of a stepped-care intervention program for perinatal depression in Nigeria [13]. The main study was a two-arm parallel cluster randomized controlled trial (RCT) comparing the intervention package for maternal depression, consisting mainly of psychological interventions, with care as usual. The formative study was aimed at establishing the feasibility of EXPONATE with the qualitative interviews conducted prior to the start of the RCT with a sample of women who had previously experienced perinatal depression.

### Study setting

The current study was conducted in Oyo State, in Southwestern Nigeria, a predominantly Yoruba speaking area. The state has 33 local government areas (LGAs), and study respondents came from the 9 LGAs selected for the main RCT study (five rural and four urban). Psychiatric care in Nigeria is mainly hospital-based and urban, although accessibility is increasing for mental health services in primary care and at maternal and child clinics (MCCs) [14]. MCCs are run by non-physician providers and midwives and are the main source of care for most low-income perinatal women, especially in rural areas. Traditional healers and faith healing practitioners are ubiquitous and provide care to many in the populace [15]. It is not unusual to have one or a few traditional healers living in a village or in large urban neighborhoods, along with local independent churches and Islamic traditionalist healers [16, 17]. Many studies of Yoruba traditional healers have been undertaken and they provide descriptions of the typical settings traditional healers work that are representative of traditional healing settings at the time of our study [16, 18]. Agara [17, pp. 114] described the typical setting of a healing church in his fieldwork in 2001, which is similar to faith healing settings in the period 2013–2014 when we conducted our study:

In all the parishes of the church, approach to healing is uniform. Means of healing…includes holy water (this is water that has been imbued with divine power to heal by prayer), amulets, prayer and fasting, sacrifices (this comes in form of fruits, clothing materials, candle, etc.), and very occasionally physical restraint. In a bare ground section within the premises of the church is the healing ground (Ile anu) which symbolically represents where Jesus Christ was born and found as a constant feature in all the parishes of the church. It is believed by members that all prayers offered on this spot would be granted.

### Participants and sampling

We aimed to sample a representative range of perspectives: patients, family caregivers and health care providers, in rural and urban settings, health care and traditional centers. Selection of study sites and participants was purposive and entailed use of maximum variation sampling (by sampling the extremes) in order to ensure we captured a broad range of perspectives. Specifically, we aimed to include in our sample patients who were first time mothers and those who were multiparous, and from divergent socioeconomic backgrounds, including both the educated and those who were illiterate, women who worked and those who were stay at home mothers, urban and rural dwellers. For family caregivers (operationalized as the patient’s spouse or a relative identified by the patient as most involved in help-seeking and care decisions), we aimed to include in our sample parents and non-parent relatives of both sexes. For health care providers, we aimed to include in our sample traditional and faith healers and physicians, and midwives and community health workers, in rural and urban settings. The study procedures were described to the facility managers in each of the MCCs, and to the proprietors of faith healing and traditional centers, and their explicit consent was obtained. Potential patient participants then were approached by research assistants and invited to take part in a study of local cultural understandings of mental problems that develop following childbirth. Study inclusion criteria included: past diagnosis of perinatal depression; family caregiver and health provider also agree to participate in the study. Exclusion criteria were patients who were acutely ill.

All the participants (n = 39) recruited for this qualitative study did not take part in the main RCT study. Participants were recruited from three target groups: (1) women (n = 14) diagnosed with perinatal depression attending MCCs, traditional and faith healing centers, and recently discharged (in the previous 12 months) from in-patient psychiatry (of the University College Hospital and the Ring Road General Hospital, both in Ibadan); (2) family caregivers (n = 14) to the women (either a mother, father, or spouse); (3) health care providers (community midwives, traditional and faith healers, general practitioners) who provided treatment to the women (n = 11). Of the 14 women, 5 (36%) were in polygamous households (or had in-laws living with them in the same house). Table 1 summarizes the characteristics of study participants.

**Table 1:** Sociodemographic characteristics of study participants

	n	%
Women (N = 14)		
Age (mean, SD) = 28 (5.59)		
Parity		
Primiparous	8	57
Multiparous	6	43
Household		
Monogamous	9	64
Polygamous	5	36
Education		
Secondary or less	8	57
Post-secondary	6	43
Location		
Hospital	8	57
Primary care center	2	14
Traditional healer	2	14
Faith healing center	2	14
Family caregivers (N = 14)		
Age (mean, SD) = 52 (13.0)		
Sex		
Female	6	43
Male	8	57
Relationship		
Spouse	5	36
Parent	9	64
Health care providers (N = 11)		
Age (mean, SD) = 48 (7.0)		
Sex		
Female	3	36
Male	7	64
Type		
Community health worker	5	45
Traditional healer	2	18
Faith healer	2	18
Physician	2	18

### Data collection

In-depth, one-on-one interviews were conducted using an adapted Yoruba-language version of the McGill Illness Narrative Interview (MINI) [19], to assess respondents’ views in regard to (1) symptoms of depression during the perinatal period, and (2) the everyday life experience of women with perinatal depression. The MINI is a semi-structured interview designed to collect qualitative data on illness experience in three main areas: (1) a temporal narrative of symptom and illness experience, organized in terms of the contiguity of events that occurred around the time of the illness (2) a description of salient personal and cultural illness prototypes related to the current health problems, based on the experience of the interviewee, the experiences of family members, friends, or others described in the media; and (3) relevant explanatory models, including cultural labels, causal attributions, expectations for treatment, course and outcome. The MINI has been widely used in cross-cultural studies of illness experience in many countries [20, 21]. A Yoruba-language version of the MINI was prepared by bilingual translators and checked for semantic equivalence by blind back-translation [22]. A copy of the adaptation of the MINI used in our study is included as online supplement (Additional file 1).

Interviews were done by facilitators trained by DG (second author), an expert in qualitative research and co-developer of the MINI. Training took place during a 3-day workshop held in Nigeria, consisting of didactic presentations and practice through role playing. Quality control was provided by a supervisor who received additional training and by DG who also reviewed the first few transcribed interviews to confirm fidelity to the protocol and provide feedback to interviewers. Interviews were conducted in strict privacy at a time and location of respondent’s choosing in English or Yoruba (the local language) according to respondent’s preference. Interviews were 1.5–2 h duration and, with respondents consent, all were audio-taped. All interviews were transcribed verbatim. Interviews in Yoruba were translated into English after transcription by bilingual research staff for subsequent analysis. Key passages were reviewed for content validity and quality (clarity, appropriateness) by bilingual co-investigators.

Informed consent was obtained at the time of the initial screening for participation in the study and again at the time of the individual in-depth interview. Ethics approval for EXPONATE was obtained from the University of Ibadan/University College Hospital Ibadan Joint Ethics Committee which is registered with the Nigerian Ministry of Health’s Research Committee and with the US Office for Human Research Protections (Federal-Wide Assurance, FWA00003094).

### Data analysis

Analysis of interview transcripts involved thematic content analysis [23, 24] with the following steps:

1. An initial open-coding phase that involved reading all interview transcripts to generate overarching themes or topic areas;
2. Further inductive thematic coding of all interview transcripts to generate subthemes organized into categories (major themes). The subthemes and categories were revised with the overarching themes identified earlier as an internal validity check;
3. Deductive (conceptual) coding for representational schemas and modes of reasoning used in respondents’ illness narratives; specifically, coding for *explanatory models*, *prototypes* and *chain complexes* elicited by the MINI [19]; and
4. Inductive thematic coding of the meanings contained in chain complexes, explanatory models and prototypes for events.

Analysis was carried out iteratively, with themes identified in the initial interviews informing the coding of later interviews. Memos detailing observations on emerging data and categories were utilized throughout the analysis. To ensure analytic rigor and representativeness of study findings, close attention was paid to identifying deviant cases and exceptions within the data set. Each transcription was initially coded by AA, and was later checked and discussed with two of the other authors (DG, LK) as a further check on validity. All further analyses were done by AA. Team members met regularly to discuss and review results of coding and instances of discrepancies were resolved through consensus. ATLAS.ti [25] was used for data storage and management.

Interpretation of themes was guided by cultural constructivist and critical anthropological perspectives to situate perinatal depression in ecosocial context as a disorder whose impact is not limited to the patient, but is embedded in webs of social relations and embodied practices enacted in specific contexts, with meanings that emerge through interactions with others [26]. Interpretation was also guided by social psychological perspectives that allow the researcher to see how social factors are structured in thought and how such factors affect depressive conflict [27]. In this paper, we present the explanatory models used by women, their family caregivers and health care providers.

## Results

### Identifying and labeling depressive symptoms and syndromes

To ascertain whether women readily recognized perinatal depression and elicit local terms for symptoms and syndromes, we asked the following questions (Items 1 and 15 on the MINI): When did you experience your health problem or difficulties for the first time? Do you have another term or expression that describes your health problem? Family caregivers and health care providers were asked the same questions but with reference to their daughter or wife or patient, respectively.

In response to the question “When did you experience your health problem or difficulties for the first time?”, study respondents talked about their “health problem” using illness terms that described a state or condition of personal and bodily distress which had some social or moral predicament as a related or constituent part. The state of distress was inseparable from the immediate social and cultural milieu. Hence, perinatal depression was inextricably linked to social and interpersonal issues. Distress states, conditions, or syndromes, identified by respondents included: *efori tulu* (unremitting headache), *irẹwẹsi ọkan*, *abisinwin* (childbirth-induced madness), *deep thought*/*thinking too much*, *sleeplessness*, *postnatal stress* and *aisan ọpọlọ* (madness) (Table 2). *Irẹwẹsi ọkan,* which translates as “blues”, but could equally be translated as “depression”, was closest to depressive disorder in current psychiatric nosology. The unity of physical, mental and social domains of experience conveyed in the use of illness terms and idioms of distress is seen in the excerpt below:

**Table 2:** Causal Attributions, Explanatory models, Idioms of Distress and Corresponding Symptoms and Signs

Causes	Mechanisms	Idioms of distress and illness terms^a^	Symptoms and signs
	[lay theory]		
1. Husband not emotionally caring	1. Thinking too much [excessive rumination]	Group 1 (milder illness): *Eforitulu*, Sleeplessness, Deep thought, Stress, Sickness after birth, Emotional deficiency, Depressive disorder, *Waku*-*gbari*	Symptoms: irrational talk and behavior, feeling down and sad, poor sleep, aimless wandering, low energy, poor appetite, weight loss, headaches
2. Problems with in-laws	2. Rise in blood pressure [local theory of stress]	Group 2 (more severe illness): *Aisan opolo*, *Abisinwin*, Madness, Mental problem, Insanity	Signs: irrational talk and behavior, looks dejected, sad, poor sleep, aimless wandering, low energy, poor appetite, weight loss, socially withdrawn, headaches, low self esteem, neglecting to care for baby
3. Spiritual attack	3. Envy by others		
4. Wanting a male child	[sorcery-witchcraft]		
5. Shock-traumatic event	4. Not ordinary causation [supernatural causation]		
6. Not resting after delivery	5. Eating in dream [mystical means]		
7. Sleeplessness	6. Sleeplessness [naturalistic causation]		
8. Medical causes (infections, pregnancy complications, difficult labor)	7. Headaches [naturalistic causation]		
	8. Eyeballs white		
	[blood shortage-balance theory]		
	9. High temperature		
	[hot and cold-balance theory]		
	10. Taboo violation [supernatural punishment]		

^a^The grouping reflects presumed causation, severity, course

**Interviewer**: Do you have another term that describes her health problem?
**Health care provider**: What I know it to be is *Abisinwin.*
**Interviewer**: What does *abisinwin* means to you?
**Health care provider**: *Abisinwin* is a Yoruba term used for the sickness [that] happens to people after giving birth which could be caused by over stress, sleeplessness, and spiritual attack.
**Interviewer**: What would really happen to those who have the illness?
**Health care provider**: They do complain of headache, behave irrationally, and sleeplessness.
  [60-year-old, female, faith healer).

The illness terms also referenced local notions of etiology and illness severity. The terms *abisinwin* and *madness* were used with attributions of more severe illness and behavioral disturbance. Women’s use of particular terms reflected severity and other features associated with the term, as in the excerpt below:

**Interviewer**: Do you have another term that describes your health problem?
**Patient**: *Efori tulu* which means persistent headache; or *irewesi okan* (depression) or shock. We can also call it *Aisan ọpọlọ* (“brain disease”).
**Interviewer**: What does *Efori tulu*, depression or *Aisan ọpọlọ*mean to you?
**Patient**: *Eforitulu* is when someone has persistent headache… it [does] not happen to only those who newly give birth but can happen to anybody anytime; depression is when someone feels disturbed and starts thinking a lot; *Aisan ọpọlọ* is when someone is not mentally okay.
  [24 years old, Para 1].

All illness terms and cultural syndromes identified by women were also used by caregivers and health providers (community health workers, traditional and faith healers). Additional illness terms used by community health workers included *depressive disorder* and *puerperal psychosis* (used interchangeably with *abisinwin*):

**Interviewer**: Do you have another term that describes her health problem?
**Health provider**: We may call it depression, or abisinwin-puerperal psychosis.
**Interviewer**: What does depression means to you?
**Health care provider**: Depression is when someone feels down, or when you feel disturbed due to some unpleasant event.
**Interviewer** (probe): What of puerperal psychosis?
**Health care provider**: I can say depression after birth, sickness that happened after birth.
**Interviewer**: What usually happens to people who have depression?
**Health provider**: They usually feel sad, feel down, they also feel discouraged, some may loss appetite for food while some may eat too much when they are depressed, some complained of severe headache.
  [45 year-old, female, community midwife].

The symptoms and descriptions of depression reported by women included sadness, irritability, loss of interest, fatigue, sleeplessness, headache, loss of appetite and weight loss, head and body crawling sensations, high temperature (body heat), rise in blood pressure, worry, excessive thinking, dejection, irrational talk and behavior (e.g. wandering, verbal and physical aggression), and neglecting to care for the baby (Table 2). Symptom descriptions such as “deep thought” (“thinking too much”), “rise in blood pressure”, “high temperature”, and “sleeplessness” were used in two different ways: (1) to refer to actual bodily and somatic phenomena, e.g., sensations of body heat or poor sleep; and (2) cultural idioms referring to a state of overwhelming stress and discomfort.

In respondents’ accounts, symptoms—whether cognitive-emotional, somatic, or behavioral—were seen as closely related to specific social and interpersonal contexts and events which gave the symptoms meaning and significance. All symptoms mentioned by the women were also mentioned by family caregivers and health care workers, and one additional symptom, social withdrawal, was only mentioned by family caregivers and health care providers. Symptoms were retrieved from quotations such as the following:

**Interviewer**: What usually happens to people who have *depression*?
**Patient**: They would misbehave, are inactive, feel dejected, they feel down, think excessively, they may loss appetite for food, and feel worried.
  [24 years old, Para 1].
**Interviewer**: What usually happens to people who have *abisinwin*?
**Family caregiver**: Such will be behaving abnormally, like talking irrationally, lose interest in doing domestic works, they would not breastfeed their baby again and they would not take care of the baby.
  [35 years old, male, spouse to patient].

### Women’s explanatory models

**Fig. 1:**
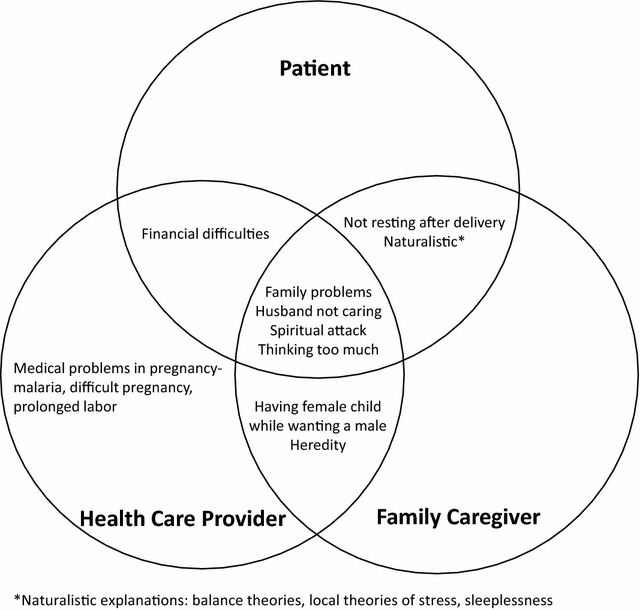
Explanatory models of patients, caregivers and health providers

Women offered multiple causal explanations, which included: *husband not caring*, *family problems*, *sleeplessness, spiritual attack*, *not resting after delivery* and *thinking too much* (see Table 2; Fig. 1). Both symptoms and causes of perinatal depression were described in relation to social suffering and interpersonal challenges in the local social worlds, linked to social roles, norms and statuses. Perinatal depression was described as linked to negative emotions, particularly regret and self-blame that women experienced when they breached social and cultural norms (e.g. refusing parents wishes in choice of marriage partners) only to later find that their choices had a poor outcome (e.g. ending up with an abusive partner):

**Interviewer**: What caused your health problem?
**Patient**: My husband was dating one woman and myself at the same time, impregnated two of us and got married to two of us without our awareness.
**Interviewer**: Are there any other causes that you think played a role?
**Patient**: My parents did not want me to be married to my husband… but I refused, so when I later saw what was going on, I felt disturbed and blamed myself.
  [24 years old, Para 1].

As shown in the excerpt above, women’s experience of illness was often presented as linked to a conflict between being self-assertive (e.g. choosing one’s own husband) yet wanting to be seen as genial, cooperative, and non-oppositional (e.g. unquestioning trust in husband). This conflict may reflect women’s awareness of the dangers of being seen as “aggressive” in a patriarchal social system coupled with the recognition that if they do not safeguard their own interests they risk having basic needs unmet. *Family problems* and *husband not caring* also spoke to this conflict linked to women’s low status in patriarchal household situations in which women’s rights and subjectivity were constrained by gender roles and norms. Lack of support from a spouse was often described in households in which the patient, her spouse, mother-in-law and rival wives (in the case of polygamous households) lived: the patient was subordinated to both her mother-in-law and rival wives, and even if he favoured her, the husband was unable to protect the patient from maltreatment by mother-in-law or rival wives:

**Interviewer**: What does depression after birth mean to you?
**Patient**: When someone feels sad, or not be in a good mood which would result to deep thought [thinking] after giving birth.
**Interviewer**: What caused your deep thought?
**Patient**: What can bring about deep thought is that when someone hurts you, you would continue to think about it.
**Interviewer**: From what you are saying it seems someone hurt you?
**Patient**: It was my mother-in-law, she is a troublesome woman.
**Interviewer**: What kind of trouble did she make for you?
**Patient**: She shouts on me without [having] offended her.
**Interviewer**: How does your husband react to it?
**Patient**: He would pet me not to be annoyed.
  [Patient, 25 years old, Para 2].

*Not resting after delivery* was a cause identified by women who attributed their illness to ignoring admonitions that they should not carry out household chores and physical exertion, and “rest” while family (in-laws in particular) attended to her and her baby. This postnatal traditional practice was understood to allow the body needed time to recuperate and restore its balance (physical and mental) after childbirth:

**Interviewer**: What caused your stress?
**Patient**: Primarily, lack of adequate rest after delivery. I was supposed to rest… [instead] I was up and doing, day and night, I did not allow my in-laws to help me, I was cooking and washing all through, some days later I had lost weight… what followed was [that] 40 days after delivery I [started] heavy bleeding and thereafter I lost my senses, [and] I started behaving like a mad person.
**Interviewer**: Are there other causes you think played a role?
**Patient**: I did not listen to my mother’s advice to rest.
  [29 years old, Para 1].

In the excerpt above, the woman linked her illness to a failure to “rest” and allow in-laws to help out, and to not heeding her mother’s advice that she “rest”.

*Spiritual attack* or “*ọwọ aiye*” (which literally translates as malign influence) explained illness in terms of the impact of supernatural influences, including witchcraft attacks, spirits or other extramundane causes that could not be explained in terms of ordinary causation (“not ordinary sickness” or “mysterious”) yet were linked to the agency of rivals in marriage and trade envious of one’s good fortune—and thus bound up with concerns about social position and unequal power relations:

**Interviewer**: Is there something happening in your life that could explain your health problem?
**Patient**: The cause is spiritual attack from my husband’s senior wife.
**Interviewer**: Can you tell me how that explains your health problem.
**Patient**: I cannot explain it, it is mysterious.
  [45 years old, Para 4].

This woman’s backtracking or reluctance to elaborate on the etiology of *spiritual attack* may reflect the risks of making interpersonal conflicts explicit as well as difficulty accepting her own aggressive impulses.

*Thinking too much* referred to rumination triggered by interpersonal difficulties or social suffering related to gender roles and social positioning, that was excessive and prolonged. This form of distress was sometimes viewed as a causal mediator between interpersonal, social and existential difficulties, and bodily responses such as *high body temperature*, *rise in blood pressure*, *sleeplessness* and *headache*:

**Interviewer**: What caused your health problem?
**Patient**: It is my thinking, I think a lot.
**Interviewer**: Are there any other causes that you think played a role?
**Patient**: I did have a rise in blood pressure, and high [body] temperature.
**Interviewer**: Why did your health problems start when it did?
**Patient**: Hmm, I remember there was a quarrel between me and my husband then, so it was the quarrel that triggered the problem.
**Interviewer**: What happened inside your body that could explain your problem?
**Patient**: I just mentioned it—I had high temperature.
  [25 years old, Para 2].

*High temperature*, *high blood pressure*, *sleeplessness*, and *white eyeballs* were symptoms or signs that conveyed women’s understanding that illness could arise from “naturalistic” causes, particularly the disequilibrium of vital body fluids and processes. These symptoms referenced local versions of balance theories of illness causation:

**Interviewer**: What caused your *abisiwin* (mental instability following childbirth)?
**Patient**: What I noticed then was that my eye balls were white.
**Interviewer**: Why did your health problem start when it did?
**Patient**: It was due to the stress and overwork.
**Interviewer**: What happened in your body to explain your health problem?
**Patient**: I was unable to sleep.
**Interviewer**: What usually happens to people who have sleeplessness?
**Patient**: The person will be weak, [and] she would not be active anymore.
  [24 years old, Para 1].

### Family caregivers’ and health care providers’ explanatory models

Family caregivers’ and health care providers’ viewed women’s illness in much the same ways as women did, as related to social suffering and interpersonal difficulties in women’s immediate social worlds, and made use of similar causal explanations (see Table 2; Fig. 1), including *husband not caring*, *family problems*, and *spiritual attack*, *thinking too much*, *high blood pressure*, *sleeplessness*, and *high body temperature*:

**Interviewer**: What caused her health problem?
**Family caregiver**: It was a frustration from the mother in law and the family. Also, there was no support from the husband.
**Interviewer**: Have you considered that she might have depression?
**Family caregiver**: Yes, she has.
**Interviewer**: What does depression mean to you?
**Family caregiver**: It is a state of feeling down as a result of lack, frustration or disappointment.
  [58 years old, female, mother to patient].

A causal explanation mentioned only by caregivers and health care providers was *having a female child while wanting a male child*, which attributed women’s illness to the dilemmas of descent and inheritance in the local patriarchal power structure, in which having a son confers privileged status on the woman in the household. Conversely, not having a male child puts the woman in a very tenuous social position:

**Interviewer**: What caused her health problem?
**Family caregiver**: It was a frustration from the mother-in-law and the family, also there were no support from the husband.
**Interviewer**: Are there other causes that you think played a role?
**Family caregiver**: She was looking for a male child because her first born was a female.
  [Mother of patient, 68 years old].
**Interviewer**: Is her health problem related to a specific event in her life?
**Health care provider**: Her husband is not happy not having a male child [and] this is an issue at home.
**Interviewer**: Can you tell us more about those events?
**Health care provider**: You see in Africa, when a man just married a new wife he spends more time with her, the old wife is not happy (and) so she causes problems at home, on top of which the purpose of marrying the new wife has not been achieved.
  [Midwife, female, 52 years old]

Apart from not having a male child as desired, caregivers and health care providers used illness explanations in similar ways to patients by invoking naturalistic causes of disequilibrium and to indicate severity.

## Discussion

This is the first study to use a qualitative methodology to investigate the lived experience and cultural interpretations of perinatal depression in Nigeria. The study identified culture-specific social and interpersonal processes that may be associated with the development of perinatal depression in sub-Saharan Africa. Women described their illness in terms of symptoms, cultural syndromes, idioms and causal explanations that linked personal bodily and emotional distress and to family predicaments. The Yoruba term *Irẹwẹsi ọkan*, which can be translated as “blues” or “depression”, was closest to depressive disorder as defined in psychiatric nosology. Illness causal explanations linked illness to naturalistic disequilibrium, interpersonal strains, and social suffering. Family caregivers and health care providers made use of many of the same terms and explanations used by women, however important differences also existed between the three groups in the explanatory models used.

Descriptions of signs and symptoms of perinatal depression provided by our study respondents are similar to those reported by studies in other African settings [28, 29], and include most symptoms listed in international criteria. Our study findings of perceived lack of support, relationship problems, and cultural factors as contributing to perinatal depression onset is in agreement with findings from previous qualitative studies of perinatal depression in other African settings [9]. Previous studies have also reported the finding “having a female child while wanting a male child”, and link it to cultural preference for male children [11], and the higher status male children confer upon the woman in matters of family inheritance [12]. Women’s inability to adhere to postnatal rites has also been identified as an important cultural factor for depression onset, especially in rural Ethiopia where the practice of postnatal period of confinement added to women’s distress because women felt unable to leave the house out of fear they would be shamed [12].

Explanatory models rooted in social and interpersonal explanations viewed women’s illness as linked to conflict between needing to be assertive to protect one’s interests but at the same time not wanting to be seen as being aggressive or oppositional in ways that contravened gender norms. This conflict was present in situations linked to disruption of norms regulating behavior in several domains: (1) hierarchical relations within the household (e.g. family problems, husband not caring, spiritual attack from rival wives, that undercut women’s agency and bring out women’s aggressive impulse); (2) perinatal rites imposed on women to safeguard their health and wellbeing (e.g. refusing to “rest” after childbirth); and (3) broader social values (e.g. giving birth to a female child that is less valued than a male child).

Social and cultural norms shape individuals thinking as to how they should be like, and people tend to live their lives by the dictates of norms and to use norms to evaluate themselves. In many parts of Africa, people are mindful that unhealthy rivalry and aggression may exist within the intimate circle of family, where ideally only solidarity and trust should reign [30, 31], and that intimates are particularly well placed to undermine one’s individual agency because they may be privy to one’s desires and vulnerabilities. As a result, individuals are socialized to have mastery of their own desires and intentions, including adroitness at “masking” or suppression of own aggressive impulses. Among the Yoruba, this value system is captured in the popular aphorism “*inu jin*”, which translates as “interiority is an unfathomable depth”. *Inu jin* is part of an ethos that views bounded interiority and internalization of aggression, as integral to character formation, and as essential to navigating the demands of social structure [32, 33].

About one-third of the women in our study lived in households that were polygamous or had in-laws residing with them in the same house, and cut across social, class, education and religious affiliations. This is consistent with findings that rates of polygamy are falling across Africa, within both rural and urban areas, for women who self-declare as “Muslim” or as “Traditional”, and among those who cannot read [34]. In patriarchal and polygamous households competition for material goods and social positioning between wives is often intense and conflictual [35, 36]. In this hierarchical social system, women need facility and adroitness to cultivate and maintain cordial relationships with rival co-wives and in-laws, if they are to access material benefits, gain social status, and exercise power as wives and mothers in the household. Women’s mastery of the expression of their own intentions and desires, including aggression, is crucial to success at these tasks of alliance-making, conflict management, and recognition within the household power structure. Internalization of patriarchal norms of femininity (e.g. being submissive and acquiescent) and motherhood (e.g. that the work of mothers is to nurture, and that respect accrues to the woman in her role as mother), and the resulting self-surveillance and self-regulation, functions to regulate women’s thinking and behaviors, and to normalize patriarchal power [37].

These processes however, also constitute “normative” or acceptable ways for women to exercise agency, express dissent and negotiate social norms, and are thus outlets for dissipating aggressive impulses, within the patriarchal structure. Thus, talk of *family problems*, *husband not caring*, *spiritual attack* and *having a female child* is a way for women to vent legitimate frustrations and draw attention to social norms and issues in their social context that undercut women’s agency. This provides a way for women and their carers to draw attention to the emotional toll of the gap between the expectations and the reality of motherhood.

Of note, women did not mention a frustrated desire for a male child as a cause of postpartum depression whereas family caregivers and health care providers did. It may be that women are unwilling to talk about this dilemma, either because doing so is socially unacceptable (and therefore potentially harmful), or because women have accepted as “natural” the tacit view that a male child is more valuable than a female one. In other words, women may have internalized patriarchal cultural norms of the centrality of male authority and domination, and the gendered roles, for example, of men leading and women supporting.

*Thinking too much* (excessive rumination) pertained to negative emotions and brooding that women experienced associated with frustrated agency and feeling not in control. Thinking too much has been recognized as a common symptom and idiom for a wide range of forms of distress across diverse cultures [38]. Rumination or brooding have a negative affect, produce more sad mood, is associated with a negative frame of mind, and leads to downward spiral of negativity which may be important mechanisms in depression.

When individuals act in a way that contradicts or conflicts with existing norms they are at risk of experiencing cultural dissonance from shame and perceived negative evaluation by others which can lead to depression [39]. Women and family caregivers (but not health care providers) in our study also linked perinatal depression to women carrying out house chores against the norm that women “rest” after delivery. In many cultures women’s reproductive behaviors are heavily controlled through social norms and ritual practices that impose normative behaviors and restrictions women are expected to follow during pregnancy and perinatal periods. These norms and practices constitute “rites of passage” [40, 41] that govern women’s behavior in their transition to a new biosocial status, their role as mothers. Rites constitute social recognition of women’s new status as mothers and confer symbolic capital, a form of power that comes with the status of motherhood [41]. Women’s symbolic capital as mothers comes from their role as caregivers with the duty of raising children and inculcating in them cultural norms and conventions which govern individuals’ behavior in society, including hegemonic norms, ensuring cultural continuity and the social reproduction of hierarchies of power [41]. Practices such as “resting” after childbirth are status markers in that they invert the power relation between a woman and her mother in-law (since in-laws now have to attend to the woman in her role as a new mother) and make manifest to all the ideologies of power associated with the status of motherhood. Consequently, when women must perform house chores despite the admonition that they desist from work as new mothers, whether because of worry about lack of support from in-laws or other constraints, they may feel exposed and shamed as unfit mothers who are undeserving of the rights and privileges that accrue to the status of motherhood. When women feel shamed in this manner, it may undercut their sense of personal agency, identity as mothers and evaluation of their own self-worth, and hence, contribute to demoralization and depression. As mentioned earlier, a similar phenomenon has been reported among postpartum women in rural Ethiopia [12], where the practice of postnatal period of confinement was found to add to women’s distress because women felt unable to leave the house out of fear they would be shamed.

Not “resting” after delivery also speaks to more pragmatic concerns, the issue of practical supports available to women postpartum the absence of which might itself be a stressor for women and contribute to the onset of perinatal depression. Our sample included women from urban and rural settings, and it may be that family configurations and available supports differ in these settings such that for urban women absent supports were related to the lack of formal public support services for new mothers, such as home visits from public health nurse, child benefit payments and maternity benefits, amongst others. Against this background, it is striking that none of the health care providers in our study identified “not resting” as a cause of postpartum depression, nor did they view the absence of support services for women as contributing to perinatal depression. The importance of public health and support services for new mothers must be understood against the background of social change, urbanization, and corresponding changes in family networks, as a source of social disadvantage and potential risk factor for perinatal depression.

Our study respondents described women’s illness using explicit bodily and psychological idioms, although illness was also seen as linked to socio-moral and personal concerns. Health providers preferred “medical” idioms rooted in biomedical explanations, while women and caregivers preferred “naturalistic” idioms that ascribed illness to imbalance in bodily processes caused by “stress”, traumatic experiences (“shock”), excessive heat, and lack of sleep. Of note, women and family caregivers made use of “naturalistic” causation explanations just as much as “supernatural” explanations of disease causation. This suggests theories of natural causation may be much more widespread and salient as lay theories of illness than recognized, and attention to such naturalistic accounts is crucial to understand women’s illness and help-seeking experiences and develop appropriate treatment interventions.

Consistent with past quantitative studies in Nigeria [3], and qualitative studies elsewhere in Africa [9, 42], respondents in our study viewed perinatal depression as both a state of psychobiological distress and the consequence of social suffering. This view of the unity of body and psyche, as interdependent with the social and cultural environment, differs from the hierarchical separation of mind and body characteristic of Western biomedical dualism [43]. However, there is recognition that a pluralistic worldview, with “flexibility, awareness, and co-existence of alternatives,” is characteristic of contemporary African experience, in part as a response to modern Western mechanistic materialism [44, p. 3].

Study respondents often used the same terms to reference cultural syndromes, idioms of distress, and causal explanations. In spite of these overlaps, important distinctions exist between these terms [45] relevant to cross-cultural work and international public health. Idioms of distress are distinguished from cultural syndromes (locally salient symptoms clusters) and explanations (etiological labels) in that idioms communicate distress without making reference to specific symptoms or syndromes; instead, they provide collective and shared ways of experiencing and talking about social suffering [38, 45, 46]. As such, idioms of distress are of special interest as they may draw attention to questions of power, such as who defines categories of distress, and patterns of help seeking [38]. Idioms also convey continuity of a person’s social and moral status in spite of illness, helping to lessen stigma, and thus also reflect notions of personhood, morality and social mobility [38].

Based on the foregoing, talk of *family problem, husband not caring*, *spiritual attack*, and *having a female child* that communicated distress without making reference to specific symptoms may be viewed as idioms of distress that communicate salient social problems and concerns related to perinatal depression in Nigerian contexts. They reflect ideologies associated with motherhood, gender relations and women’s rights and agency. They also highlight links between help seeking and the orientation of local systems of healing.

Our study has some limitations, including a relatively small sample, and possible selection bias introduced by recruiting only women in treatment (in hospital, health centers, faith and traditional centers). However, our findings are consistent with those of previous studies of perinatal depression in Africa, and extends them in important ways. The present study sheds light on the expressions of and explanations for perinatal depression in Nigeria, with implications for understanding help-seeking, treatment, and prevention. Eliciting the perspectives of patients, family caregivers and health care providers allowed us to identify commonalities and divergences in the ways that perinatal depression is explained. Interpretation of themes using cultural constructivist and ecosocial perspectives allowed us elaborate processes mediating cultural risk factors for perinatal depression.

## Conclusions

In Southwestern Nigeria, mental health problems in the perinatal period are recognized both as discrete illness entities and as reflections of suffering related to gender roles and social norms. Women’s illness was described using bodily and psychological idioms rooted in naturalistic causation, and sociomoral idioms rooted in supernatural causation. Perinatal depression was also linked to conflicts between women’s need to be self-assertive while wanting not to be seen as being aggressive. These conflicts may hinder women’s sense of agency and efficacy, leading to rumination, negative self-appraisal and shame from perceived negative evaluation by others. Clinical assessment and interventions as well as public health prevention strategies for perinatal depression in global mental health need to consider local social contexts and meanings of depression, which can be explored with the methods described in this study.

## Additional file

**Additional file 1.** The McGill Illness Narrative Interview (MINI)-adapted version used in EXPONATE interviews.
