# Supplementary material for: Perinatal depression in Nigeria: perspectives of women, family caregivers and health care providers
Source: Int J Ment Health Syst. 2017 Apr 17;11:27. doi: 10.1186/s13033-017-0134-6 (PMC5392941; doi:10.1186/s13033-017-0134-6)
Supplement: Supplementary file 1 — Additional file 1. The McGill Illness Narrative Interview (MINI)-adapted version used in EXPONATE interviews. [file 13033_2017_134_MOESM1_ESM.docx]

Adaptation Version of the McGill Illness Narrative Interview (MINI)

**AGE:**

**SEX:**

**LEVEL OF EDUCATION:**

**RELIGION:**

**LOCATION of Interview:**

Question 1: When did you [family member/patient] experience your [her] health problem or difficulties for the first

time?

Question 2: We would like to know more about your experience. Could you tell us when you realized you [she]

had this health problem?

Question 3: Can you tell us what happened when you [she] had her health problem?

Question 4: If you [she] went to see a helper or healer of any kind, tell us about your

[her] visit and what happened afterwards.

Question 5: If you [she] went to see a doctor, tell us about your [her] visit to the doctor/

hospitalization and about what happened afterwards.

Question 6: Did you [she] have any test or treatment for her health problem?

Question 7: In the past, did you ever had a health problem that you considered similar

to your [her] current health problem?

Question 8: Did a person in your family ever experience a health problem similar to

yours [hers]?

Question 9: Did a person in your social environment (friends or work) experience a

health problem similar to yours [hers].

Question 10: Have you ever seen, read or heard on television, radio, in a magazine, a

book or on the internet of a person, who had the same health problem as

yours [hers]?

Question11: Do you have another term or expression that describes your [her] health problem?

Question 12: According to you, what caused your [her] health problem (list primary

causes)?

Question 13: Why did your [her] health problem start when it did?

Question 14: What happened inside your [her] body that could explain your [her] health

problem?

Question 15: Did something happening in the family, work or social life that could

explain your [her] health problem?

Question 16: Can you tell me how that explains your [her] health problem?

Question 17: Have you considered that you [she] might have [use illness term provided

by respondent to question 12]?

Question 18: What does [illness term provided by respondent to question 12] mean to

you?

Question 19: What usually happens to people who have [use illness term provided by

respondent to question 12]?

Question 20: What is the best treatment for a person who has [use illness term

provided by respondent to question 12]?

Question 21: How do other people react to someone who has [use illness term provided

by respondent to question 12]?

Question 22: Who do you know has had [use illness term provided by respondent to

question 12]?

Question 23: Is your [her] health problem linked or related to specific event that

occurred in your [her] life?

Question 24: During the visit to your health care provider for your [her] health problem,

what did your health provider tell you that your [her] problem was?

Question 25: Did your health care provider give you [her] any treatment, medicine or

recommendations to follow?

Question 26: How are you dealing with each of this recommendation?

Question 27: Are you able to follow that treatment (recommendation or medicine)?

Question 28: What made that treatment work well?

Question 29: What made that treatment difficult to follow or work poorly?

Question 30: What treatment do you expect to receive for your [her] health problem that you did not receive?

Question 31: What other therapy, treatment, help or care have you sought out?

Question 32: What other therapy, treatment, help or care would you like to receive?

Question 33: Has your [her] health problem changed the way you [she] lives?

Question 34: How has your [her] health problem changed the way you [she] feels or

think about yourself [herself]?

Question 35: How has your [her] health problem changed the way you look at life in

general?

Question 36: How has your [her] health problem changed the way that others look at

you [her]?

Question 37: What has helped you [her] through this period in your [her] life?

Question 38: How have your family or friends helped you through this difficult period of

your life?

Question 39: How has your spiritual life, faith or religious practice helped you go

through this period of your life?

Question 40: Is there anything else you will like to add?
